# A Crowd Disaster Study: The Itaewon Seoul Crush

**DOI:** 10.7759/cureus.68811

**Published:** 2024-09-06

**Authors:** Alexander H Chang, Soon-Joo Wang, Aakash Anandjiwala, Edbert B Hsu

**Affiliations:** 1 Global Health, Center for Global Emergency Care, Johns Hopkins University, Baltimore, USA; 2 Emergency Medicine, College of Medicine, Hallym University, Seoul, KOR; 3 Medicine, William Carey University College of Osteopathic Medicine, Hattiesburg, USA; 4 Disaster Medicine, Center for Global Emergency Care, Johns Hopkins University, Baltimore, USA

**Keywords:** crowd crush, crowd disaster, crush injuries, human stampede, seoul halloween crowd crush

## Abstract

The recent Halloween crowd crush incident in Itaewon, Seoul, Korea, highlights a woeful inadequacy in our collective knowledge about crowd disasters. Underscored is a lack of detailed information on the causes of death and the injuries sustained. While traumatic asphyxiation has been widely implicated as the primary cause of death, the wider spectrum of injury patterns and their causative mechanisms remain poorly identified. Challenges to advancing our understanding of crowd disasters include limited and restricted access to official reports, incomplete documentation, and reliance on unofficial sources for information. There is a need for targeted research to better understand crowd dynamics that lead to injuries. Future directions should prioritize better interdisciplinary collaboration, improved data sharing, and computer simulations to model real-world events. Further inquiry into human stampedes and crowd crushes, such as the Itaewon incident, is urgently needed to prevent similar tragedies; focusing on the types and mechanisms of injuries is a crucial first step to enhancing emergency preparedness and disaster response.

## Editorial

Crowd disasters such as human stampedes and crowd crushes pose a significant public health challenge. Catastrophic events can occur with little warning whenever dense groups gather in restricted spaces. A sudden surge can ripple through the crowd, generating intense, deadly compressive forces. In other instances, human stampedes can trigger a domino effect, causing victims to fall and be trampled underfoot. These incidents, often in connection with sporting events, music concerts, religious gatherings, and political demonstrations, as well as other spontaneous or unplanned events, reveal the inherent risks associated with any dense gathering [1].

The public health ramifications of crowd disasters and the resultant impact on healthcare systems need to be carefully documented and studied. Without planning and coordination, local healthcare facilities can be rapidly overwhelmed. In the aftermath of crowd disasters, morbidity and mortality often occur within a short time period. Crowd disasters not only cause physical trauma but also leave lasting psychological effects on survivors, witnesses, and the general population. Furthermore, such disasters continue to occur as evidenced by a stampede at a religious gathering in northern India in 2024, killing at least 121 people [2].

Despite the substantial media coverage crowd disasters typically attract, there remains a paucity of studies on crowd disasters. Data regarding the types of trauma sustained are limited, and the precise etiologies of mortality in crowd-related incidents remain largely unexamined. In this analysis, we take a closer look at the Seoul Halloween Crowd Crush, which occurred on October 29, 2022, resulting in hundreds of casualties, including at least 159 deaths and 196 injuries [3].

**Figure 1:**
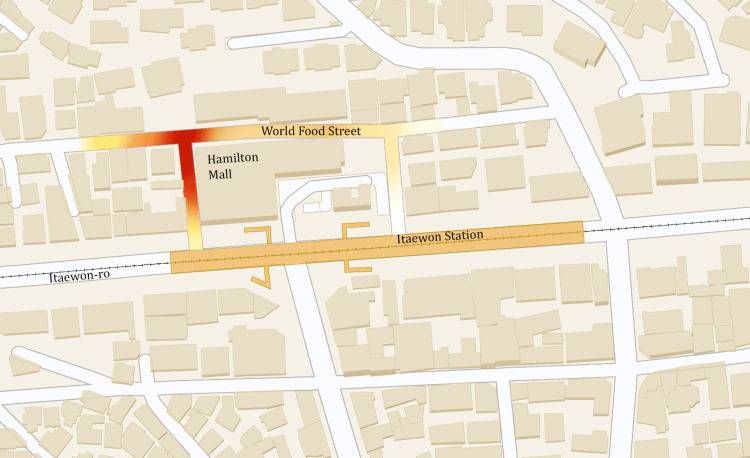
Map of the Itaewon crowd crush incident

On October 29, 2022, crowds of over 100,000 packed the narrow streets and alleyways during Halloween festivities in the Itaewon district of Seoul, South Korea [4]. The Itaewon district is a popular nightlife area with trendy nightclubs, restaurants, and bars, attracting both locals and tourists alike. Around 10:00 PM, frantic emergency calls overwhelmed dispatch centers as a devastating crush enveloped hundreds in scenes of chaos with many trapped in an alley bottleneck (Figure 1) [4]. The ensuing crowd disaster was a Class IV stampede with over 300 casualties [5]. Rescues were arduous with significant challenges in reaching and aiding victims [4]. Later investigations revealed that calls hours earlier to authorities for assistance went unheeded [4]. Lack of crowd control, adequate policing, and escape routes contributed to the worst peacetime tragedy in South Korea since the sinking of the Sewol ferry in 2014 [6]. While crowd disasters receive intensely focused media coverage, far fewer details are readily available regarding the types of injuries sustained, mechanisms of injury, or causes of death among stampede victims. Estimates of crowd size and crowd density often lack precision [4]. Furthermore, findings from official reports are often not publicly accessible due to ongoing investigations and political, legal, or security concerns. These are missed opportunities to apply and leverage valuable experience.

Scant literature exists on mechanisms of injury during stampedes. A single published case report from the Itaewon crowd disaster details the medical presentation of a 27-year-old survivor [7]. Initially, experiencing severe pain, paresthesia, and paresis in the lower back and extremities, with elevated creatine kinase and liver enzyme levels, the female survivor was diagnosed with sciatic and common peroneal neuropathy accompanied by rhabdomyolysis [7]. Far more than individual case studies are needed. In these situations, few autopsies are typically performed, and their reports are often not released. This highlights the need for more transparency and targeted research. A collective evolved understanding of injuries during crowd disasters would be indispensable to emergency managers, first responders, and designated receiving hospitals.

Reports from other crowd disasters suggest that the compressive forces leading to traumatic asphyxiation are the predominant cause of death during crowd disasters. Traumatic asphyxiation is associated with prolonged force applied to the torso or other parts of the body. Intense pressure from tightly packed crowds restricts movement and chest excursion. One study found that for an adult male, 2550 ± 250 N (260 ± 26 kg) of static chest pressure is sufficient to cause a flail chest [8]. Accounts of forces generated by a crowd strong enough to bend steel railings (approximately 4500 N) have been reported [1]. A thorough examination of the dynamics of force generation and its various implications during crowd disasters is needed. Compressive forces on the torso can cause blockage of venous outflow resulting in edema and cyanosis. Other notable post-mortem findings include eye hemorrhages and petechiae across the upper body [5]. In the Itaewon crush, many of the victims who succumbed were disproportionately female and of smaller stature, suggesting that even lower thresholds of force can prove to be lethal [8]. Individual factors such as age, gender, physical fitness, and pre-existing health conditions all likely impact the chances of survival.

Due to deadly compressive forces during a crowd disaster, victims may often expire while crushed upright, only collapsing to the ground when pressures abate. Compression from front to back, causing ventilatory failure, is perhaps more deadly than lateral compression that spares the lungs and rib cage [8]. Anecdotal reports from some Itaewon survivors suggest that they were saved by turning their bodies to escape direct crush forces [9]. Crowd crush injuries encompass a wide range extending beyond traumatic asphyxiation. Myocardial infarction and other direct blunt trauma injuries leading to the crushing of intrathoracic organs have been identified as other causes of death [10]. During human stampedes, as in the Itaewon crowd disaster, individuals who have fallen are frequently unable to get up. These downed victims may be trampled underfoot, sustaining crush injuries, brain and internal organ damage, fractures, and neurovascular compromise. Still, there remains a conspicuous scarcity of literature examining the full spectrum of injuries that can occur in crowd disasters.

The Itaewon stampede and other crowd disasters can be especially difficult to study. Rarely do events described in the literature provide nuanced details of specific injuries or injury mechanisms. Official reports are often not shared, as in this case, due to ongoing legal, political, or security considerations. Parsing extensive media coverage can be informative but wrought with limitations. The fragmented nature of media reports skirts the description of technical details such as mechanisms of injury and causes of death, knowledge of which is essential to future emergency management planning and efforts. Personal and eyewitness accounts posted on social media may reflect internal biases or lack verification. More importantly, the inherent nature of crowd disasters involves tremendous human loss; balancing sensitivity to trauma-informed care and the necessary respect for privacy adds to the challenge of obtaining critical information from victims and witnesses. Our current modest understanding of crowd disasters reflects these realities.

Numerous questions related to injuries in the context of crowd disasters demand answers. The characteristics and distribution of injuries sustained during a crowd disaster vary widely, and determining the percentage of salvageable injuries and the likelihood of survival during transport to a trauma center remain critical areas where our knowledge is lacking. Exploring the mechanisms of injuries that occur during a crowd disaster and the implementation of real-time restrictions on pedestrian traffic once established thresholds are exceeded are crucial for improving safety measures. To effectively design measures that protect individuals in crowded environments, it is essential to understand how localized crowd size and density are correlated with deadly and near-miss events. In addition, systematically capturing and sharing information from a crowd disaster with authorities, emergency managers, event planners, first responders, and local hospitals is vital for improving response efforts and preventing future incidents.

One promising area of research involves advancements in computer simulations to develop virtual environments that accurately replicate real-world scenarios and explore how various factors, such as crowd size, layout, and exit locations, influence crowd behavior. Simulations can effectively identify potential bottlenecks and high-risk areas that could support real-time crowd monitoring and management systems. Systems incorporating surveillance cameras and sensors to detect crowd density and movement patterns could be used to provide early warnings to authorities. Coupled with crowd behavior prediction programs, proactive measures could be taken to prevent crowd crushes before they occur.

Interdisciplinary collaboration will be essential for advancing crowd crush research. Experts from fields such as traumatology, psychology, sociology, engineering, computer science, and design need to work together to develop comprehensive approaches to crowd management. By gaining insights into human behavior, cultural factors, and social dynamics, crowd control strategies can be put in place that are not only effective but also adaptable for different events.

The Itaewon disaster on October 29, 2022, in Seoul, Korea, is a stark reminder of the devastation that can be wrought by unchecked crowds. In this tragedy, a critical density was breached, leading to a catastrophic crowd crush with over 300 casualties. The primary cause of fatalities in this disaster was largely attributed to intense compressive forces that resulted in traumatic asphyxiation and severe internal injuries. Many victims were unable to survive due to the overwhelming pressure exerted by the densely packed crowds, underscoring the impact of such forces on the human anatomy.

Further research into crowd disasters, particularly focusing on types and mechanisms of injuries, is necessary to advance our collective understanding. Identifying risk factors, elucidating crowd behavior, and exploring how crushes evolve are essential for developing proactive measures to prevent future crowd disasters. The Itaewon disaster highlights the need for improved real-time monitoring of crowd density, early warning systems, and defined triggers for the implementation of safety protocols to prevent similar tragedies. By integrating the lessons learned from Itaewon and other crowd disasters, we can take significant steps toward mitigating risks inherent in mass gatherings.
